# Transient attention does not alter the eccentricity effect in estimation of duration

**DOI:** 10.3758/s13414-023-02766-6

**Published:** 2023-08-07

**Authors:** Alina Krug, Lisa Valentina Eberhardt, Anke Huckauf

**Affiliations:** Department of General Psychology, Institute of Psychology and Education, Ulm University, 89069 Ulm, Germany

**Keywords:** Duration estimation, Time perception, Visual periphery, Eccentricity, Transient attention, Exogenous cueing, Spatial attention

## Abstract

Previous research investigating the influence of stimulus eccentricity on perceived duration showed an increasing duration underestimation with increasing eccentricity. Based on studies showing that precueing the stimulus location prolongs perceived duration, one might assume that this eccentricity effect is influenced by spatial attention. In the present study, we assessed the influence of transient covert attention on the eccentricity effect in duration estimation in two experiments, one online and one in a laboratory setting. In a duration estimation task, participants judged whether a comparison stimulus presented near or far from fixation with a varying duration was shorter or longer than a standard stimulus presented foveally with a constant duration. To manipulate transient covert attention, either a transient luminance cue was used (valid cue) to direct attention to the position of the subsequent peripheral comparison stimulus or all positions were marked by luminance (neutral cue). Results of both experiments yielded a greater underestimation of duration for the far than for the near stimulus, replicating the eccentricity effect. Although cueing was effective (i.e., shorter response latencies for validly cued stimuli), cueing did not alter the eccentricity effect on estimation of duration. This indicates that cueing leads to covert attentional shifts but does not account for the eccentricity effect in perceived duration.

**Supplementary information:**

The online version contains supplementary material available at 10.3758/s13414-023-02766-6.

## Introduction

A seemingly simple everyday task like catching a ball appears to be a quite complex action when considering that this requires a synchronization of perceptual, motor and mental functions. Even within the perceptual domain a synchronization between spatial and temporal processing across the visual field is required to successfully accomplish the task. Strikingly, subjective perception of duration is distorted in the visual periphery. Given that the distribution of spatial covert attention also differs across the visual field (e.g., LaBerge & Brown, 1986, 1989) and attentional shifts can alter perceived duration (e.g., Seifried & Ulrich, 2011; Yeshurun & Marom, 2008), this highlights the importance of investigating peripheral time perception with regard to spatial attentional shifts. In the present study we investigate how transient spatial attention modulates duration estimation across retinal eccentricity.

### Effects of retinal eccentricity on estimation of duration

A majority of studies investigating the influence of retinal eccentricity on temporal processing (e.g., Carrasco et al., 2003) and subjective duration (e.g., Aedo-Jury & Pins, 2010; Jovanovic & Mamassian, 2019; Kliegl & Huckauf, 2014) suggest different types of temporal processing in the visual periphery (but see Westheimer, 1983). Studies examining duration estimation of peripheral stimuli have shown that the duration of stimuli in retinal eccentricity is typically underestimated (i.e., Aedo-Jury & Pins, 2010; Jovanovic & Mamassian, 2019; Kliegl & Huckauf, 2014).

In 2010, Aedo-Jury and Pins asked observers to compare the duration of two stimuli in the form of empty intervals whose onset and offset were marked by two short visual flashes. Stimuli were positioned at 6° of eccentricity above and below a central fixation cross. The horizontal position of the comparison stimulus varied between 0° and 48° of eccentricity. Results showed an increase in time compression with increased stimulus eccentricity. Equalizing potential differences in the visibility for stimuli across the visual field, the authors found this underestimation of stimulus duration only for magnocellular-biased but not for parvocellular-biased stimuli. Thus, they attribute the increased time compression with increased eccentricity in duration estimation to differences in basic visual functions.

In a series of five experiments, Kliegl and Huckauf (2014) investigated the influence of stimulus eccentricity on estimation of duration using simple stationary black disks as stimuli. Participants were instructed to compare the duration of a foveally presented standard stimulus with a subsequent comparison stimulus presented at 3°, 6° or 9° of eccentricity. Replicating the findings of Aedo-Jury and Pins (2010), the results indicated an underestimation of duration with increasing stimulus eccentricity. Importantly, when controlling for differences in basic visual functions by equalizing the size of the cortical projection areas for peripheral stimuli through m-scaling (Experiments 4 and 5), temporal underestimation of peripheral stimuli persisted. This suggests that the effect might not be due only to the physiologically inhomogeneous structures of the visual system determining performance differences within the visual field.

To sum up the above reviewed studies, there seem to be some (i.e., Aedo-Jury & Pins, 2010) but not entirely convincing evidence (i.e., Kliegl & Huckauf, 2014) that differences in basic visual processing circuits between central and peripheral vision, such as the size of cortical projection areas or processing in distinct visual pathways, contribute to the effect of underestimation of duration in the visual periphery. Thus, it is likely that other factors in visual processing contribute to the effect.

Another important difference between central and peripheral vision is the distribution of spatial attention. Attention is assumed to be maximal at focus and gradually decreases with increasing stimulus eccentricity (e.g., LaBerge & Brown, 1986, 1989; Yeshurun & Carrasco, 1998). This may account for the observed eccentricity effect in duration estimation: More peripheral stimuli receive a lower degree of spatial attention, which leads to a shortening of perceived stimulus duration. Thus, when spatial attention is shifted toward peripheral stimulus positions, duration underestimation at these positions should be reduced. Using durations of up to 2 s, Jovanovic and Mamassian (2019) in fact provided evidence that attention modulates the differences in temporal processes between central and peripheral stimuli. Following up on the attentional account of the eccentricity effect, they replicated the eccentricity effect on duration estimation using clock-like moving stimuli. Furthermore, they examined the distribution of visual attention using a probe to alter visual attention. They observed that besides the effect of eccentricity, stimuli were also reported as being earlier if they were presented near attended features in the visual scene. This suggests that exogenous attention affects the estimation of duration. However, Jovanovic and Mamassian (2019) have used durations of up to 2 s in their study, an interval that allows not only for covert attentional shifts, but also for overt eye movements. Hence, it is still unclear whether spatial attention underlies eccentricity effects in the estimation of duration.

Taken together, subjective duration as measured in a variety of tasks and experimental set-ups declines with increasing retinal eccentricity. One mechanism potentially underlying such effects of retinal eccentricity on the estimation of duration is the distribution of visual attention across the visual field. In the current study, we investigate the impact of attentional shifts on the eccentricity effect in subjective duration by means of transient cueing.

### Effects of attention and spatial cueing on estimation of duration

While research on the role of stimulus eccentricity on perceived duration is scarce, a large body of research has focussed on the role of attention mechanisms. Attention plays a key role in a variety of time-perception models, including pacemaker-counter models such as the attentional-gate model by Zakay and Block (1995, 1997) or the striatal beat frequency model by Matell and Meck (2000, 2004). In general, research shows that distracting attention from time decreases perceived duration whereas perceived duration increases when attention is guided towards time. For instance, studies investigating the effect of divided attention show that distracting attention from a temporal task decreases the accuracy of duration estimates (for a review, see Block et al., 2016; Brown, 2008) and compresses perceived time when performing a nontemporal secondary task in a dual-task paradigm (Brown, 2008; Hemmes et al., 2004).

Further, studies investigating the effect of spatial attention show that pre-cueing a stimulus location leads to an overestimation of duration compared to stimuli that were not preceded by a cue. Thus, when attention is allocated to a stimulus, its duration is perceived as being longer. This holds for endogenous cues (e.g., Enns, Brehaut, & Shore, 1999; Mattes & Ulrich, 1998) as well as for exogenous (e.g., Seifried & Ulrich, 2011) and transient cues (Yeshurun & Marom, 2008). In the present study we use transient cueing to avoid overt allocation of attention.

In a series of experiments, Seifried and Ulrich (2011) investigated the effect of exogenous visual attention on perceived duration of brief visual stimuli. They used peripheral luminance cues. In the first experiment, a standard stimulus consisting of a white dot was presented in the centre of the screen. A grey square frame was positioned to the right and left from the screen centre. The cue consisted of a brief brightening from grey to white (53 ms) of one of the frames after presenting the standard stimulus (100 ms or 300 ms). After an inter-stimulus interval (ISI) of 53 ms, the comparison stimulus, consisting of either the letter X or the letter O was flashed within either the cued (validly cued trials) or the uncued frame (invalidly cued trials), with a validity of 50%. The squared frames remained visible throughout the whole trial, therefore avoiding potential temporal biases by keeping the local information surrounding the stimuli comparable between the valid and invalid cueing conditions. This ensures that the results do not reflect the combined duration of cue and target. Participants judged validly cued stimuli to be longer than invalidly cued stimuli. In addition, reaction times for validly cued trials were shorter compared to invalidly cued ones. The authors replicate the effect that attended stimuli are perceived as longer in five subsequent experiments using different cueing displays and measurement methods. Thus, the results provide strong evidence that directing spatial attention by exogenous cueing prolongs perceived duration when controlling for local information surrounding the stimulus.

Yeshurun and Marom (2008) investigated the effect of transient spatial attention on perceived duration of stimuli at various eccentricities. In two experiments, participants compared the duration of two successively presented brief stimuli (23–94 ms and 23–165 ms, respectively) in the form of black discs presented at 2°, 5°, 8° or 12° of eccentricity above or below, left or right of fixation. One of the discs was preceded by an attentional peripheral cue (attended disc), a green horizontal bar presented for 50 ms above the position of the subsequently presented disc. The other disc was preceded by a neutral cue (neutral disc), a green circle in the centre of the screen that was presented for 50 ms that did not indicate the position of the subsequent disc. Replicating the effects of Seifried and Ulrich (2011), the results indicate that the attended disc was perceived as being longer compared to the unattended disc, suggesting that transient attention prolongs perceived duration. Yeshurun and Marom (2008) do not present statistics of the effect of eccentricity on duration estimation and only mention that duration judgements did not vary as a function of stimulus eccentricity. Moreover, contrary to Seifried and Ulrich (2011), local information surrounding the stimuli was not controlled in the two experiments from Yeshurun and Marom (2008) described above. Therefore, combining the duration of cue and target, in the sense of summing up their durations, may have occurred. To address this argument, the authors conducted a third experiment. Here, they applied a multibar cueing display similar to that of Seifried and Ulrich (2011), but tested only at an eccentricity of 5°. Also, by controlling the local information surrounding the stimulus that way, the duration of the disc preceded by an attentional cue was perceived as being longer. Taken together, exogenously and transiently cued stimuli are perceived as longer. But, it is still unclear how transient attention alters effects of retinal eccentricity in duration estimation when information surrounding the stimulus is controlled for.

### The present study

If a gradient of decreased attention accounts for the temporal underestimation of peripheral stimuli as we suggest here, one would expect that the eccentricity effect is reduced when validly precueing the target position. Hence, we assume that with increased eccentricity stimulus duration is underestimated only for neutrally cued stimuli, not for validly cued ones where attention is deployed to the stimuli. In the present study, we investigated the influence of covert shifts of spatial attention on the eccentricity effect in duration estimation of short stimuli in an online (Experiment 1) and a laboratory eye-tracking experiment (Experiment 2). In a duration estimation task, participants judged whether a comparison stimulus with varying duration presented at 3° or 9° of eccentricity was shorter or longer than a centrally presented standard stimulus with a constant duration. A cueing display avoiding spatial interaction was used to manipulate transient covert attention. Covert attention was either directed to the position of the subsequent peripheral comparison stimulus by a transient luminance cue (valid cue) or did not convey information regarding the position (neutral cue). Using the neutral cue, we expected the eccentricity effect found by Aedo-Jury and Pins (2010) and by Kliegl and Huckauf (2014) to be observed here as well. If the effect of duration underestimation with increasing stimulus eccentricity is influenced by covert shifts of spatial attention, the eccentricity effect should be reduced or even disappear when using valid cues.

## Experiment 1: Online study

### Methods

#### Participants

Participants were recruited via the institutional participant pool management system SONA and institutional mailing lists. All subjects gave informed consent to participate. Subjects participating via SONA received partial course credits for their participation. Participants were naïve regarding the purpose of the study, and all reported normal or corrected-to-normal vision.

Eighty-six participants completed the online experiment; 30 male, 56 female, no diverse sex was reported. The reported mean age was 29.85 years (*SD*_age_ = 13.81). All participants gave informed consent before participation. The experimental procedure was conducted in agreement with guidelines set out in the Declaration of Helsinki.

#### Apparatus and stimuli

The experiment was run as an online experiment. It was implemented using PsychoPy3, v2020.1.3 experiment builder (Peirce et al., 2019) and hosted on Pavlovia.org. Participants accessed the experiment from their preferred web browser via a link provided by the experimenter, and therefore conducted the experiment from their own devices.

To determine the screen size and resolution of each participant’s monitor the virtual chinrest method described by Li, Joo, Yeatman, and Reinecke (2020) was used. The virtual chinrest works via a card task for estimating the pixel density in pixels per mm: Participants were instructed to adjust the size of a credit card image on the screen to the size of a real credit card or card of equal size. Then, pixel density was calculated as the ratio between the on-screen size of the card image in pixels and the physical size of the credit card in mm. This enables presentation of the stimuli at a constant size, independent of the individual screen characteristics. To ensure a constant viewing distance throughout the experiment, participants were instructed to keep an arm’s length of distance between themselves and the monitor. Afterwards, each participant’s individual viewing distance was calculated by measuring the distance from a fixation point to the entry point of the eye’s blind spot area, as described by Li et al. (2020). That is, while covering their right eye, participants fixated a black cross in the right screen half with the left eye. A red dot was moving away from fixation, and participants were asked to press the space bar as soon as the red dot seemed to disappear, indicating the entry point of the blind spot area. The individual viewing distance was estimated by calculating the distance between the centre of the fixation cross and the red dot using knowledge of the entry point of the human blind spot area (13.5°) and trigonometry.

A fixation cross in the centre of the screen was set to 0.5° × 0.5° in size, according to the measurements of screen size and resolution and the viewing distance using the virtual chinrest method (Li et al., 2020). The luminance of the programmed stimuli was measured exemplarily by a CAST MAVOLUX 5032B USB luxmeter on the screen of a Lenovo ThinkPad T460s Ultrabook (1,920 × 1,080 px). The central fixation cross (0.5° × 0.5°) was set to black (lum = 0.3 cd/m^2^; RGB: 0, 0, 0). The stimuli consisted of black discs of 0.8° diameter (lum = 0.3 cd/m^2^; RGB: 0, 0, 0), which were presented on a dark grey background (lum = 34.3 cd/m^2^; RGB: 128, 128, 128), either in the centre of the screen (standard stimulus) or at a near or far eccentricity from fixation left or right on the horizontal meridian (comparison stimuli). These eccentricities were set to 3° (near) and 9° (far) of eccentricity in both the left and the right visual field, which results in four potential positions where the comparison stimulus might appear.

**Fig. 1:**
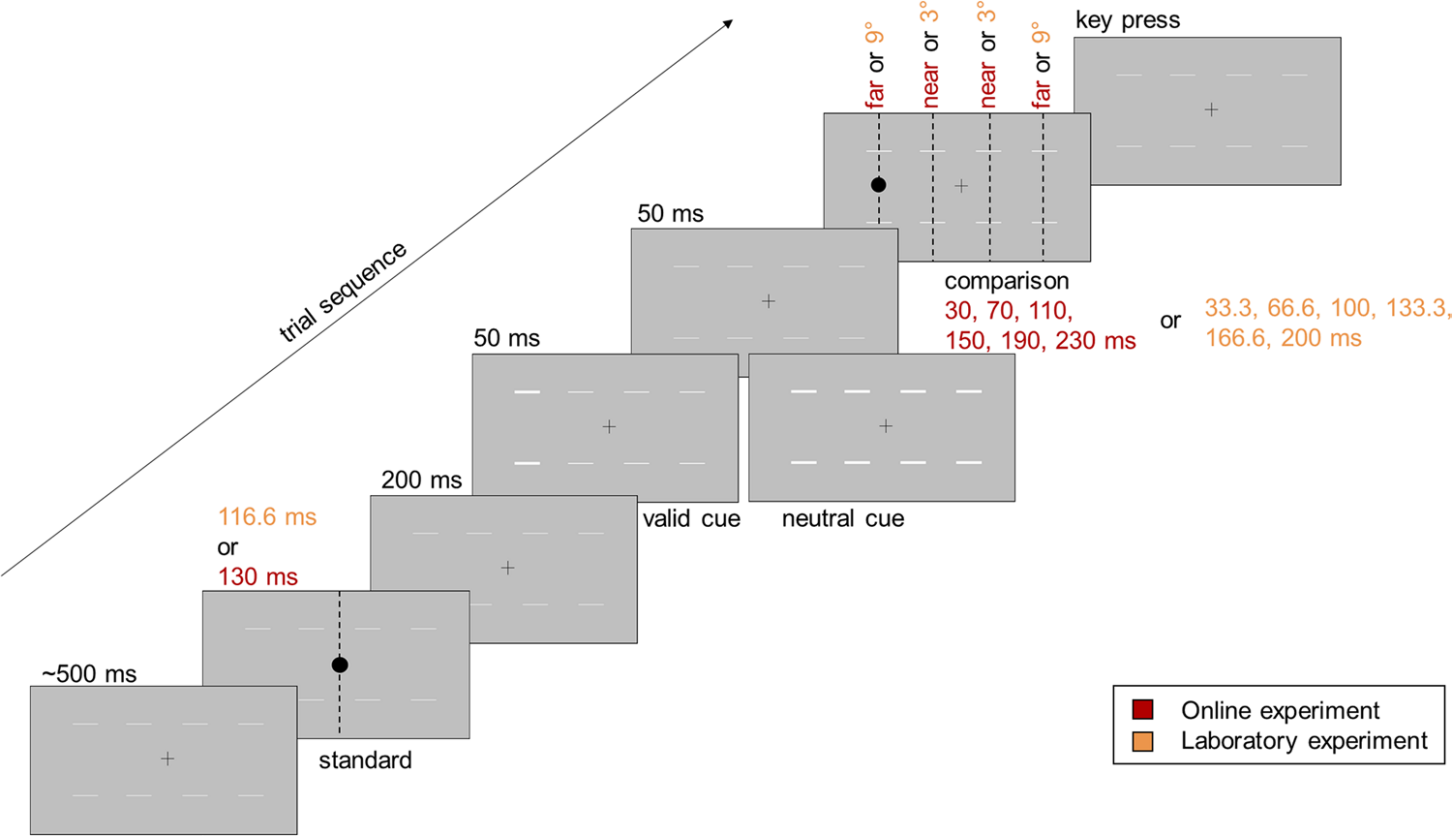
Illustration of a trial sequence. Stimulus durations and positions implemented in Experiment 1 are depicted in red, durations used in Experiment 2 are depicted in orange. The comparison stimulus was preceded by either the valid or the neutral cue and was presented in either the near or the far eccentricity condition. Vertical dotted lines indicate the stimulus position on the screen and were not presented throughout the trial

Although conventional screens typically refresh at 60 Hz (e.g., Anwyl-Irvine et al., 2021; Bridges et al., 2020; Sauter et al., 2020), the frame rate of the screens actually used by the participants cannot be obtained by PsychoJS, the online counterpart of PsychoPy (Bridges et al., 2020). Since it is impossible to determine the stimulus duration precisely in online experiments (Bridges et al., 2020), the duration of the stimuli was set to the intended duration throughout the experiment, which was 30, 70, 110, 150, 190 or 230 ms for the comparison stimuli. Compared to Kliegl and Huckauf (2014), slightly longer stimulus durations were chosen since dropped frames can occur during online studies (e.g., Anwyl-Irvine et al., 2021; Bridges et al., 2020). Multibar cues (similar to Yeshurun & Marom, 2008; see Fig. 1) were used and the cueing display was designed with recourse to the set-up used by Seifried and Ulrich (2011). Light grey bars (lum = 89,8 cd/m^2^; RGB: 191, 191, 191) with a size of 2.53° (width) × 0.175° (height) were programmed to appear 1.26° above and 1.26° below each of the four possible positions of the comparison stimulus. The cue was a change in the brightening of the bars from grey to white (lum = 185.3 cd/m^2^; RGB: 255, 255, 255). Figure 1 illustrates the cueing display as well as the sequence of a trial.

#### Design and procedure

The experimental design comprised four factors varying within participants: cueing (2: valid, neutral), eccentricity (2: near, far), visual field (2: left, right), and duration (6: 30, 70, 110, 150, 190, 230 ms), resulting in 48 cells. Each cell was repeated ten times, resulting in a total of 480 trials. Trials were presented within five experimental blocks consisting of two repetitions per cell, which were presented in random order. Except for the first block, in which ten practice trials were added at the beginning, participants completed five randomly chosen practice trials before each of the four remaining blocks. Participants were reminded to keep an arm’s length distance from the monitor before each experimental block started to ensure a constant viewing distance.

The experiment lasted about 30 min. After giving informed consent, the subjects answered questions regarding their age, sex and eyesight. Afterwards, the screen size and resolution were determined and participants’ viewing distance was estimated by using the virtual chinrest method described above (Li et al., 2020). Participants were instructed to keep their gaze on the fixation cross throughout each trial and started by completing ten practice trials. Each trial started with a fixation cross displayed for a mean intended duration of 500 ms (*SD* = 25 ms, min = 100 ms, max = 900 ms). Then, the standard stimulus was presented in the screen centre for 130 ms. After 200 ms, the cue was presented for 50 ms. In the neutral cue condition, the horizontal bars at each of the four possible positions of the comparison stimulus changed their colour from grey to white. In the valid cue condition, only the bars at the position of the subsequent comparison stimulus changed colour, with the other bars remaining grey. After an ISI of 50 ms (cf. previous studies by Seifried & Ulrich, 2011, and Yeshurun & Marom, 2008), the comparison stimulus was presented. The comparison stimulus was displayed for 30, 70, 110, 150, 190 or 230 ms at either the near or far eccentricity from fixation in the left or right visual field. The participants' task was to decide whether the comparison stimulus was longer than the standard stimulus. Responses were to be entered via the keyboard; “yes” was to be indicated by pressing “L” with the right middle finger, “no” by pressing “N” using the right index finger. After the key press a new trial was initiated. The procedure is illustrated in Fig. 1.

#### Data analysis

Data analysis was performed with Spyder 5.1.5 under Python 3.9.7 (Van Rossum & Drake, 2009) and JASP 0.16.4.0 (JASP Team, 2023). The point of subjective equality (PSE) was calculated for each participant and condition (cue × eccentricity). It was determined by fitting a logistic function to the observed relationship between the response “longer” and the duration of the comparison stimulus (cf. Aedo-Jury & Pins 2010; Kliegl & Huckauf, 2014). The PSE indicates the duration of the comparison stimulus that is equally likely to be estimated as “shorter” or “longer” and therefore to be subjectively as long as the standard stimulus. In case the estimated duration matches the actual duration of the comparison stimulus, the PSE equals the duration of the standard stimulus. Higher values indicate that the duration of the comparison stimulus is underestimated; that is, it has to be prolonged to be judged as equally long as the standard stimulus. Lower values indicate an overestimation of the duration of the comparison stimulus.

The data sets of 49 subjects with correct response rates in all experimental conditions above chance (> 60%) were included in further analysis to ensure a reliable calculation of the respective PSE values (cf. Kliegl & Huckauf, 2014). *R*^*2*^ values were calculated for each condition to evaluate the goodness of fit. Tukey’s (1977) boxplot method to identify outliers for symmetric and skewed data was used to examine the distribution of *R*^*2*^ values in the respective experimental conditions. The method calculates the first (Q1, 25th percentile) and third (Q3, 75th percentile) quartile of a given data set as well as the interquartile range, which is the difference Q3−Q1. Extreme outliers are defined as values outside three times the interquartile range above or below Q3 or Q1, respectively (Dawson, 2011; Hoaglin et al., 1986). Forty-six participants (23 males, 23 females; *M*_age_ = 29.74 years, *SD*_age_ = 13.69) were included in the statistical analysis since their* R*^*2*^ values in all conditions were not identified as extreme outliers. Mean *R*^*2*^ was .948 (min = .812; max = .999).

Further, reaction times were compared to investigate whether cueing affected performance. Average reaction times were obtained by calculating the median reaction times of all trials for each participant and condition (cue × eccentricity).

A 2 × 2 repeated-measures ANOVA with cue (neutral, valid) and eccentricity (near, far) as repeated-measures factors was conducted for PSE values and reaction times using JASP 0.16.4.0 (JASP Team, 2023).

### Results

**Fig. 2:**
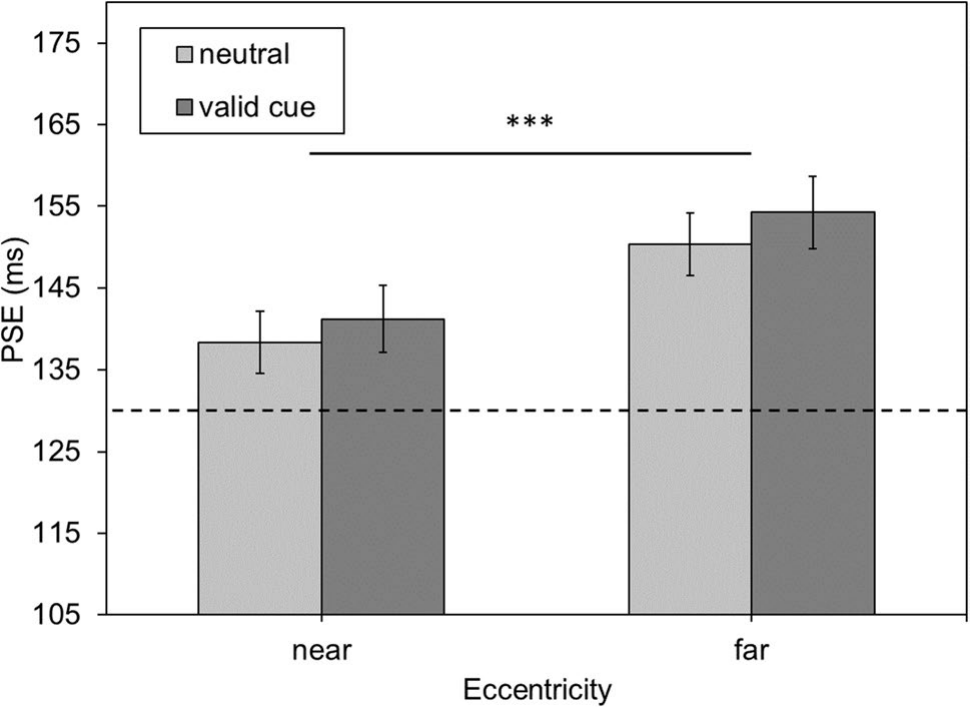
Mean point of subjective equality (PSE) values in milliseconds as a function of the eccentricity of the comparison stimulus (near, far) and the two cueing conditions (valid vs. neutral cue). Error bars represent the standard error (SE). The dotted horizontal line represents the duration of the standard stimulus. *Note:* * *p* < .05, ** *p* < .01, *** *p* < .001

Mean PSE values are depicted in Fig. 2. With neutral cues, the PSE was *M*_neutral/near_ = 138.352 ms (*SE*_neutral/near_ = 3.762 ms) for the near and *M*_neutral/far_ = 150.382 ms (*SE*_neutral/far_ = 4.127 ms) for the far eccentricity. With valid cues, the PSE was *M*_valid/near_ = 141.202 ms (*SE*_valid/near_ = 3.820 ms) for the near and *M*_valid/far_ = 154.239 ms (*SE*_valid/far_ = 4.406 ms) for the far eccentricity. The ANOVA confirmed a significant main effect of eccentricity, *F*(1,45) = 17.356, *p* < .001, ${\eta }_{p}^{2}$ = .278. Neither the main effect of cue nor the interaction between cue and eccentricity were significant (*p* = .130 and *p* = .711, respectively). Hence, these data replicate the eccentricity effect in duration estimation in that there is an underestimation of durations with increasing eccentricity.

**Fig. 3:**
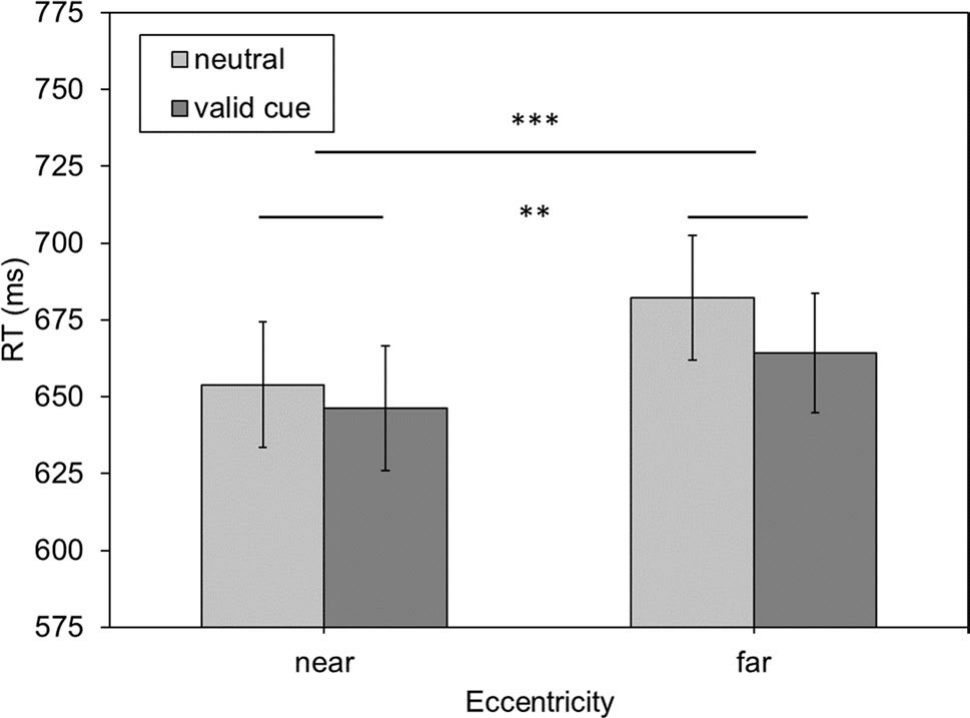
Mean reaction time (RT) in milliseconds as a function of eccentricity of the comparison stimulus (near, far) and the two cueing conditions (valid vs. neutral cue). Error bars represent the standard error (SE). *Note:* * *p* < .05, ** *p* < .01, *** *p* < .001

Contrary to expectations, cueing did not alter the eccentricity effect in duration estimation. In order to see whether cueing did produce any effect on performance at all, mean reaction times were compared (see Fig. 3). For near stimuli, reaction times to validly cued stimuli are *M*_valid/near_ = 646.311 ms (*SE*_valid/near_ = 20.320 ms) and *M*_neutral/near_ = 653.942 ms (*SE*_neutral/near_ = 20.473 ms) for neutral cues. For far stimuli, reaction latencies for valid cues are *M*_valid/far_ = 664.269 ms (*SE*_valid/far_ = 19.420 ms) and *M*_neutral/far_ = 682.114 ms (*SE*_neutral/far_ = 20.262 ms) for neutral cues. The repeated-measures ANOVA yielded a significant main effect of eccentricity, *F*(1,45) = 29.166, *p* < .001, ${\eta }_{p}^{2}$ = .393, and of cue *F*(1,45) = 11.551, *p* = .001, ${\eta }_{p}^{2}$ = .204. There was no significant interaction *F*(1,45) = 1.430, *p* = .238, ${\eta }_{p}^{2}$ = .031.

### Interim discussion

The results of the online experiment replicate the effect of duration underestimation with increasing eccentricity as reported by previous studies (Aedo-Jury & Pins, 2010; Kliegl & Huckauf, 2014; Jovanovic & Mamassian, 2019). Hence, examining the subjective duration of short durations of up to a quarter of a second is still possible when going online. Regarding our main question at issue, Experiment 1 investigated how the distribution of visual spatial attention contributes to this eccentricity effect. Results indicate that cueing did not affect the PSE and therefore did not contribute to the eccentricity effect. In order to see whether cueing did produce any effect on performance at all, mean reaction times were compared since studies investigating covert attentional shift using exogenous or transient cues typically show a decrease in reaction latencies for valid compared to neutral cues (Cameron, Tai, & Carrasco, 2002; Carrasco, 2011; Posner, 1980; Seifried & Ulrich, 2011; Yeshurun & Carrasco, 1999). The present results also demonstrate this effect. However, most striking regarding the reaction latencies obtained in the present online study is that they exceed those usually obtained in studies investigating duration estimation (Seifried & Ulrich, 2011) or covert attentional shifts by means of exogenous or transient cueing (Carrasco & Yeshurun, 2009; Posner, 1980). Since an overall increase in reaction time is not necessarily true for online studies (e.g., Anwyl-Irvine et al., 2021; Sauter et al., 2022), it must be considered that participants did not work at their performance maximum during the online experiment. Therefore, we replicated the experiment in the laboratory.

## Experiment 2: Laboratory eye-tracking study

### Methods

#### Participants

Thirty-nine subjects with normal or corrected-to-normal vision (ten male, 28 female, *M*_age_ = 23.1 years, *SD*_age_ = 3.04) were recruited via SONA and institutional mailing lists. One participant did not provide information regarding age and sex. Subjects could either receive partial course credits or a payment of 12.50 € for participation. All subjects gave informed consent before participation and were naïve with regard to the study objective. The experimental procedure conducted was in line with guidelines set out by the Declaration of Helsinki.

#### Apparatus and stimuli

Experiment 2 was also implemented using PsychoPy3 v2020.1.3 experiment builder (Pierce et al., 2019) and run in a dark and sound-proof laboratory of Ulm University. Stimuli were presented on a 60 × 34-cm BenQ XL2720-B monitor (4096 × 2160 px) running with 60 Hz. A head-chinrest ensured a constant viewing distance of 96 cm at which the display subtended 34° × 20° of visual angle. An Eyelink^®^ 1000 Plus controlled for saccadic eye movements by monocularly tracking the right eye. Two Logitech Multimedia Speakers Z200 informed participants about saccadic eye movements that took place during stimulus presentation. A standard keyboard was used as a response device.

The stimulus material used in Experiment 2 did not differ from Experiment 1, with the following exceptions: Luminance of the grey background (lum = 97 cd/m^2^), black fixation cross, standard and comparison stimuli (lum = 0.4 cd/m^2^) and light grey bars (lum = 194 cd/m^2^) was measured by a CAST MAVOLUX 5032B USB luxmeter on the screen. Again, the cue was a change in brightening of the bars from grey to white (lum = 292 cd/m^2^). In Experiment 2, the duration of the standard stimulus was set to 116.6 ms, the comparison stimulus was presented for 33.3, 66.6, 100, 133.3, 166.6 or 200 ms.

#### Procedure and design

Design and procedure of the experiment remained the same, except for the following changes: The participants only performed ten practice trials in the beginning and a 9-point calibration and validation of the eye-tracker was conducted before each of the five experimental blocks. The experiment lasted about 1.25 h. The sequence of a trial did not change in Experiment 2 (see Fig. 1). Again, participants were instructed to indicate whether the comparison stimulus was longer than the standard stimulus, this time by pressing the “down” arrow with the right index finger for “no” and the “up” arrow with the right middle finger for “yes”.

In case the eye position deviated more than 0.5° of eccentricity from the fixation cross on the horizontal meridian during stimulus presentation, the respective trial was repeated at the end of the five experimental blocks. In this case participants heard a 400-Hz feedback tone for 250 ms via the speakers at the end of the trial reminding them to fixate the screen centre.

#### Data analysis

As in Experiment 1, PSE values were calculated for each participant and condition. The data of 31 participants were included in further analysis due to a correct response rate of > 60% in all experimental conditions (cf. Kliegl & Huckauf, 2014). Again, the boxplot method (Tukey, 1977) was used to identify extreme outliers regarding the goodness of fit as indicated by *R*^*2*^. Based on this criterion, the data of three participants were discarded. Mean *R*^*2*^ was .960 (min = .873; max = .995).

Data analysis was performed with a 2 × 2 repeated-measures ANOVA with cue (neutral, valid) and eccentricity (3°, 9°) as repeated-measures factors for PSE values and reaction times using JASP 0.16.4.0 (JASP Team, 2023).

### Results and discussion

**Fig. 4:**
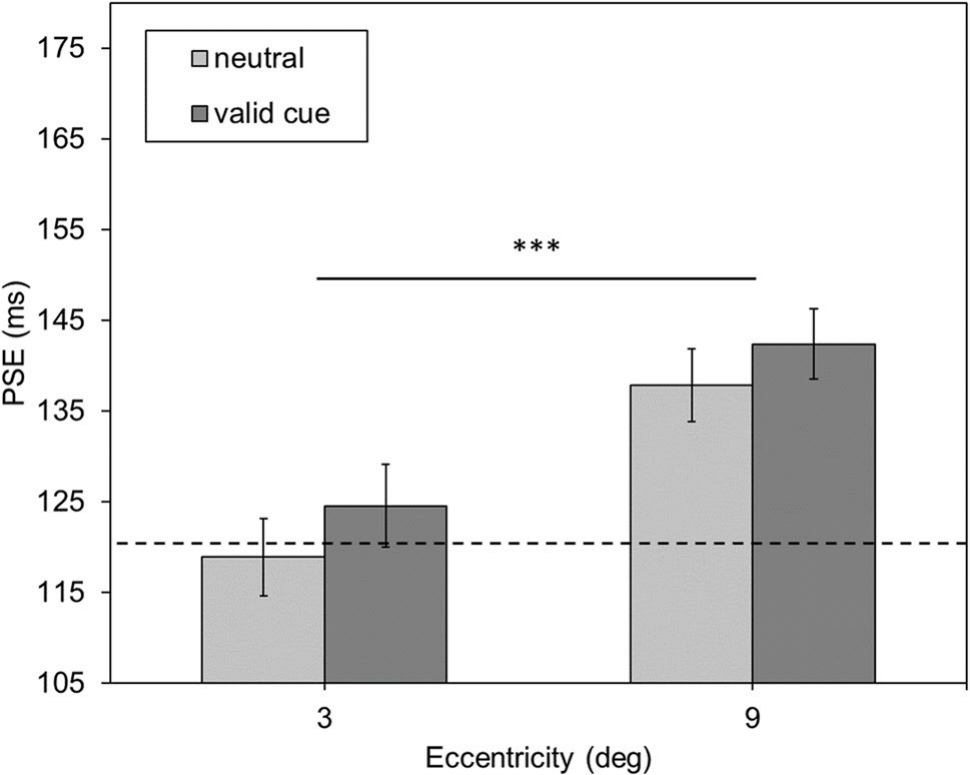
Mean point of subjective equality (PSE) values in milliseconds as a function of the eccentricity of the comparison stimulus (3°, 9°) and the two cueing conditions (valid vs. neutral cue). Error bars represent the standard error (SE). The dotted horizontal line represents the duration of the standard stimulus. *Note:* * *p* < .05, ** *p* < .01, *** *p* < .001

Figure 4 displays the mean PSE values. As in Experiment 1, for 3° of eccentricity results yielded *M*_neutral/3°_ = 118.894 ms (*SE*_neutral/3°_ = 4.238 ms) for the neutral cue and *M*_valid/3°_ = 124.555 ms (*SE*_valid/3°_ = 4.581 ms) for the valid cue condition. For 9° of eccentricity mean PSE values were *M*_neutral/9°_ = 137.845 ms (*SE*_neutral/9°_ = 4.036 ms) and *M*_valid/9°_ = 142.400 ms (*SE*_valid/9°_ = 3.914 ms) for the neutral and valid cue conditions, respectively.

Replicating Experiment 1, the ANOVA showed a significant effect of eccentricity on PSE values *F*(1,27) = 28.668, *p* < .001, ${\eta }_{p}^{2}$ = .515, but neither a significant main effect of cue *F*(1,27) = 3.164, *p* = .087, ${\eta }_{p}^{2}$ = .105, nor an interaction *F*(1,27) = 0.119, *p* = .733, ${\eta }_{p}^{2}$ = .004. Hence, cueing did not affect duration estimation, as in Experiment 1. Moreover, as in Experiment 1, the results of Experiment 2 successfully replicate the effect of duration underestimation with increasing stimulus eccentricity (cf. Aedo-Jury & Pins, 2010; Kliegl & Huckauf, 2014; Jovanovic & Mamassian, 2019).

**Fig. 5:**
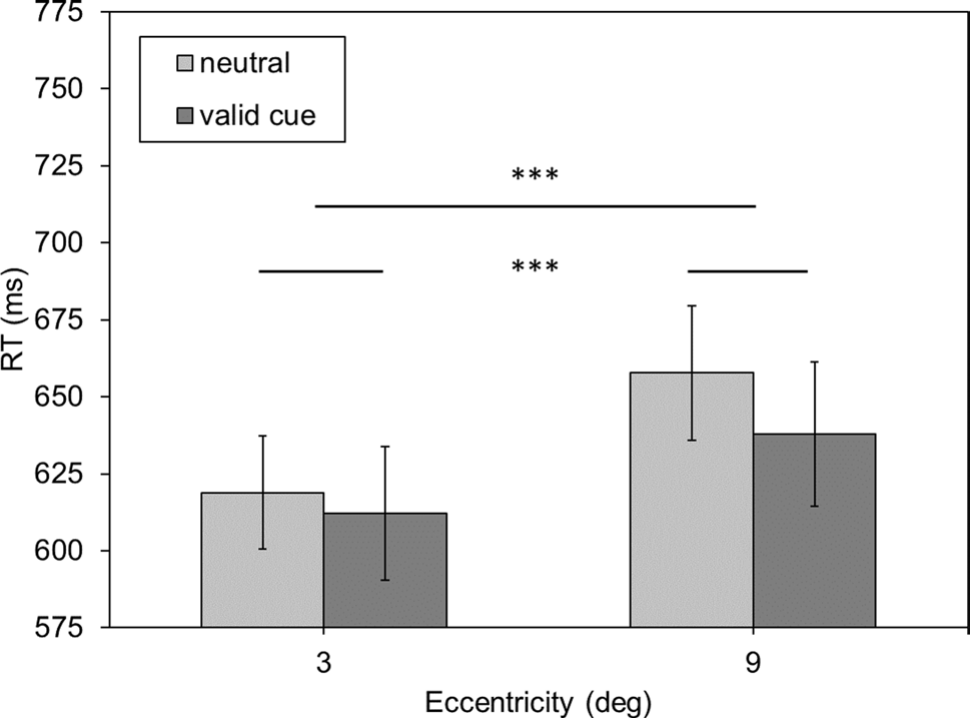
Mean reaction time (RT) in milliseconds as a function of eccentricity of the comparison stimulus (3°, 9°) and the two cueing conditions (valid vs. neutral cue). Error bars represent the standard error (SE). *Note:* * *p* < .05, ** *p* < .01, *** *p* < .001

Again, mean reaction times (Fig. 5) were analysed to determine whether the cue affected performance in the laboratory study. At an eccentricity of 3°, reaction time for the valid cue was *M*_valid/3°_ = 574.005 ms (*SE*_valid/3°_ = 17.842 ms) and *M*_neutral/3°_ = 589.531 ms (*SE*_neutral/3°_ = 15.412 ms) for the neutral cue. At 9° of eccentricity for validly cued stimuli, reaction time was *M*_valid/9°_ = 595.691 ms (*SE*_valid/9°_ = 18.650 ms) and *M*_neutral/9°_ = 617.196 ms (*SE*_neutral/9°_ = 18.743 ms) for neutrally cued ones. As in Experiment 1, the ANOVA showed significant main effects of eccentricity, *F*(1,27) = 17.504, *p* < .001, ${\eta }_{p}^{2}$ = .393, and of cue, *F*(1,27) = 25.547, *p* < .001, ${\eta }_{p}^{2}$ = .486, and no interaction, *F*(1,27) = 0.493, *p* = .488, ${\eta }_{p}^{2}$ = .018, was found. That is, reaction latencies were significantly shorter when validly cueing the stimulus position compared to a neutral cue condition, thus showing an unequivocal cueing effect. However, there was no interaction, which means that transient covert attention, although effective here, did not alter the eccentricity effect in duration estimation, neither in the PSE nor in the corresponding reaction times.

## General discussion

In the current experiment, we investigated duration estimation in the periphery using an online experimental set-up (Experiment 1) as well as a laboratory eye-tracking set-up (Experiment 2). Both experiments successfully replicate effects of stimulus eccentricity on the duration estimation of short stimuli (see Aedo-Jury & Pins, 2010; Kliegl & Huckauf, 2014; Jovanovic & Mamassian, 2019). Additionally, the eccentricity effect was obvious in higher reaction time latencies with increased eccentricity, also replicating known effects (e.g., Carrasco & Yeshurun, 1998; LaBerge & Brown, 1986). Directing attention was also successfully manipulated by the transient luminance cue as indicated by shorter reaction latencies when validly cueing the stimulus position compared to a neutral cue condition in both experiments, replicating previous work (e.g., Cameron, Tai, & Carrasco, 2002; Yeshurun & Carrasco, 1999; Seifried & Ulrich, 2011) showing an unequivocal cueing effect. The main goal was to investigate whether and how transient cueing alters effects of retinal eccentricity on duration estimation when information surrounding the stimulus is controlled for. However, again clearly shown in both experiments, cueing did not affect the PSE. That is, although the cue did indeed lead to a covert attentional shift that did speed up the responses, it did not influence the duration judgement nor did it interact with the eccentricity of the stimuli.

### Transient attention does not affect duration estimation of peripheral stimuli

Although clear effects of cueing have been shown, and although clear effects of eccentricity on duration estimation and reaction latencies have also been replicated, cueing did not interact with eccentricity in two experiments, even when controlling for eye movements. Hence, we have to conclude that the effect of eccentricity on duration estimates at issue in the current setting is not due to covert shifts of spatial attention.

Former studies investigating the effect of cueing on duration estimation found that precueing the stimulus location leads to stimuli being judged as longer (e.g., Mattes & Ulrich, 1998; Seifried & Ulrich, 2011; Yeshurun & Marom, 2008). In the present study, we did not find an effect of transient cueing on duration estimation, neither in the online setting of Experiment 1, nor in the well-controlled laboratory setting in Experiment 2. Previous studies report a prolongation of duration estimates by cueing (Seifried & Ulrich, 2011; Yeshurun & Marom, 2008). At this point, we can only speculate why cueing did not alter the eccentricity effect in duration estimation or affect duration estimates at all in the present study. When interpreting differences between effects on duration estimation in various studies, the specific properties of the implemented cueing paradigm have to be carefully taken into consideration.

In the present study, the cueing display ensured that temporal integration between cue and target, which could account for a temporal prolongation, was not possible. Also, Seifried and Ulrich (2011) carefully designed a cueing display in which local information surrounding the stimuli was controlled for. They observed that exogenous attention prolongs subjective duration, but tested only one position in the periphery. Yeshurun and Marom (2008) also found a temporal prolongation by precueing across various eccentricities, but could not fully rule out for various eccentricities that this is due to a local temporal integration of cue and target. In the present study, when ensuring that local information around the stimuli remains constant over time and testing several eccentricities, no temporal prolongation of subjective duration is found. Hence, in the light of these results it appears unlikely that the effects are associated with a spatio-temporal integration of cue and target durations.

Another important difference in the design of the presentation display between the present study and the study by Seifried and Ulrich (2011) is the possibility to execute eye movements. Covert attentional shifts are closely linked to saccadic eye movements, as they precede the initialization of these after presenting a peripheral stimulus (Deubel, 2008; Deubel & Schneider, 1996). Since most of our durations do not allow for saccadic eye movements after the cue in both experiments (Mayfrank et al., 1987) and trials in which a saccade occurred were not included in the data analysis of Experiment 2, overt attentional shifts cannot underlie the effects at issue. However, this is not the case for the study by Seifried and Ulrich (2011), who found a temporal prolongation, since at least the comparison durations presented subsequent to the long standard stimulus allowed for the execution of eye movements after the cue. Previous studies have shown that saccadic eye movements affect duration estimates (e.g., Morrone et al., 2005, Yarrow et al., 2001); specifically, goal-directed saccades towards a stimulus prolongs its perceived duration (e.g., Yarrow et al., 2001). Thus, planning and/or execution of saccadic eye movements may account for the duration prolongation found by Seifried and Ulrich (2011). This highlights the importance of controlling for saccadic eye movements in future studies with regard to peripheral duration estimation. Taken together, the present results indicate that the distribution of visual attention across the visual field does not determine differences in estimation of duration.

Taking into account literature on the functions of parvocellular and magnocellular ganglion cells showing their distribution on the retina differs between centre and periphery (Lee et al., 2010; Masri et al., 2020) rather suggests a complex interrelationship between spatial attention and duration estimation. Since we think this is worth being investigated directly in future studies, we elaborate on the idea in detail below.

While the parvocellular pathway is responsible for high spatial acuity as well as green and red colour vision (Lee et al., 2010), the magnocellular pathway mainly serves motion detection (Kaplan & Shapley, 1986), thus requiring a high temporal resolution. Reviewing evidence on magnocellular and parvocellular processing suggests that while the former drives peripheral duration estimation (Aedo-Jury & Pins, 2010), transient attention only facilitates stimuli processed in the latter. Indications of this are found in the large body of research showing that spatial attention enhances spatial resolution (e.g., Yeshurun & Carrasco, 1998; for reviews see Carrasco & Yeshurun, 2009, and Anton-Erxleben & Carrasco, 2013) and impairs temporal resolution (e.g., Montagna & Carrasco, 2006). Studies examining stimuli directly biased towards either magnocellular or parvocellular processing yielded differential effects of transient covert attentional shifts on magnocellular-mediated task performance (e.g., Yeshurun & Levy, 2003; Yeshurun & Sabo, 2012). For example, a study by Yeshurun and Sabo (2012) showed that precueing a target location led to better task performance only for stimuli biased towards parvocellular processing, while there was no difference in task performance for stimuli biased towards magnocellular processing. In contrast, Yeshurun and Levy (2003) showed that precueing the target location even reduced performance in a temporal acuity task mediated by the magnocellular pathway. The authors argue that attention facilitated the activation of parvocellular neurons and inhibited magnocellular neurons at the same location, leading to a reduced temporal resolution in the temporal acuity task (cf. Peñaloza & Ogmen, 2022). Similar results were obtained for other temporal resolution-dependent tasks, such as flicker rate discrimination (Montagna & Carrasco, 2006) and motion direction discrimination (Pavan et al., 2022).

Taken together, research shows that transient shifts of covert attention increase performance only for tasks mediated by the parvocellular pathway, while the performance for tasks mediated by the magnocellular pathway is either unaffected by spatial attention or even impaired.

Indications that magnocellular processing also plays a key role for the perceived duration of peripheral stimuli can be derived from the study from Aedo-Jury and Pins (2010). Based on their finding that processing differences in the magnocellular pathway are responsible for the effect of duration underestimation with increased eccentricity and that transient attention facilitates stimulus processing mediated by the parvocellular, but not magnocellular, pathway (Yeshurun & Levy, 2003; Yeshurun & Sabo, 2012), it is plausible that shifts in spatial attention would not influence the processing of peripheral stimuli, as investigated in the present experiment. This is supported by a post hoc analysis of the difference limen (DL) as a measure of temporal sensitivity (Bausenhart et al., 2018) we performed to investigate whether shifts in spatial attention affected temporal resolution in the present study. While temporal sensitivity was lower for peripheral compared to more central stimuli (cf. Yeshurun & Levy, 2003), cueing did not lead to an overall decrease in temporal sensitivity in both experiments (see Online Supplemental Material (OSM)). This corresponds to the results from Seifried and Ulrich (2011), who also did not find a significant effect of cueing on the DL in a similar duration discrimination task.

Although the stimuli were not designed to explicitly bias processing towards the magno- or parvocellular pathway, the results obtained in the present study support this notion. Hence, we might suspect that transient attention facilitates processing mediated by the parvocellular pathway (Yeshurun & Levy, 2003; Yeshurun & Sabo, 2012), whereas the effect of duration underestimation of peripheral stimuli is mediated by the activity in the magnocellular pathway (Aedo-Jury & Pins, 2010).

### Psychophysical investigation of duration estimation online is possible

The effect of cueing was clearly shown in the laboratory setting of Experiment 2 as well as in the online setting of Experiment 1. Acquiring behavioural data in online studies has gained popularity over the past decade (Sauter et al., 2020). Various paradigms in experimental psychology, including psychophysical tasks such as Stroop, Eriksen-flanker, visual search, priming, attentional blink (Crump et al., 2013; Semmelmann & Weigelt, 2017), task switching, Simon task, and Posner cueing (Crump et al., 2013) have been investigated in online studies. By replicating the eccentricity effect in duration estimation in Experiment 1, we confirm for the first time that online settings are also feasible to study the subjective duration of short stimuli. This is especially noteworthy since stimulus timing is crucial for duration estimation studies, and is less precise in online studies (Anwyl-Irvine et al., 2021; Bridges et al., 2020). Mean DL values were calculated post hoc to compare temporal sensitivity (Bausenhardt et al., 2018) between the online setting of Experiment 1 and laboratory setting of Experiment 2 (see OSM). Results indicate a lower temporal sensitivity for the online *M*_Experiment 1_ = 41.406 ms (*SE*_Experiment 1_ = 2.604 ms) compared to the laboratory study *M*_Experiment 2_ = 32.657 ms (*SE*_Experiment 2_ = 2.207 ms). Yet, the precision achieved in the online experiment seems to be sufficient for a reliable fitting procedure and measurement of the PSE.

While tools such as the virtual chinrest method may be feasible to counteract the limited monitoring of experimental conditions such as stimulus size and location (Li et al., 2020), limited participant monitoring remains a potential problem with online studies (Sauter et al., 2020). Compared to Experiment 2, only about half of the participants achieved correct response rates above chance in the online setting, indicating much more noisy data. Besides technical issues (e.g., more variance in timing, in screen size, in screen resolution, in brightness, in reflections, etc.; Bridges et al., 2020), this might also be due to less clear instructions since there are less opportunities to clarify requests or give direct feedback (Sauter et al., 2020). The lack of monitoring also may have caused participants to work below their performance maximum, leading to higher reaction times in the online experiment (also see Seifried & Ulrich, 2011). Taken together, studying duration estimation online is possible, yet inefficient.

### Conclusion

Taken together, the results of the present study support the robustness of the eccentricity effect in duration estimation (Aedo-Jury & Pins, 2010; Kliegl & Huckauf, 2014; Jovanovic & Mamassian, 2019), even when investigated in a less controlled online environment (Experiment 1). Therefore, the present results add evidence to the growing body of literature showing that conducting psychophysical studies online is a feasible but perhaps inefficient alternative to laboratory-based studies, at least in terms of examining duration estimation. Moreover, the results show that although cueing accelerated duration judgements, it did not alter the temporal underestimation for large relative to small eccentricities. Therefore, we conclude that attentional shifts as a consequence of transient spatial cueing do not contribute to the eccentricity effects on duration estimation. This is in line with evidence showing differential effects of transient attention on magnocellular processing, which seems to drive the effect of duration underestimation with increasing eccentricity. Ultimately, in the present study covert attentional shifts towards the visual periphery did not account for differences in duration estimation between central and peripheral stimuli.

### Supplementary Information

Below is the link to the electronic supplementary material.

Supplementary file1 (DOCX 120 KB)
