# Supplementary material for: Transient attention does not alter the eccentricity effect in estimation of duration
Source: Atten Percept Psychophys. 2023 Aug 7;86(2):392–403. doi: 10.3758/s13414-023-02766-6 (PMC10806013; doi:10.3758/s13414-023-02766-6)
Supplement: Supplementary file 1 — Supplementary file1 (DOCX 120 KB) [file 13414_2023_2766_MOESM1_ESM.docx]

Supplement

## Analysis of the difference limen in Experiment 1

This appendix contains the analysis of the difference limen (DL) for *n* = 46 participants in Experiment 1. A 2 × 2 repeated measures ANOVA with cue (neutral, valid) and eccentricity (near, far) as repeated measures factors was conducted on DL values using JASP 0.16.4.0 (JASP Team, 2023).

The difference limen (DL) was calculated post-hoc to investigate temporal sensitivity. Mean values for each condition are shown in Figure S1. For the near eccentricity condition mean DL values are *M_neutral/near_* = 38.494 ms (*SE_neutral/near_* = 2.651 ms) for the neutral cue and *M_valid/near_* = 41.981 ms (*SE_valid/near_* = 3.281 ms) for the valid cue condition, respectively. In the far eccentricity mean DL values for neutrally cued stimuli are *M_neutral/far_* = 42.438 ms (*SE_neutral/far_* = 2.539 ms) and *M_valid/far_* = 42.710 ms (*SE_valid/far_* = 3.065 ms) for validly cued stimuli. A repeated measures ANOVA revealed no significant main effect of eccentricity *F*(1,45) = 2.459, *p* = .124, $\eta_{p}^{2}$ = .052 and of cue *F*(1,45) = 1.185, *p* = .282, $\eta_{p}^{2}$ = .026 and no significant interaction *F*(1,45) = 1.975, *p* = .167, $\eta_{p}^{2}$ = .042.

*
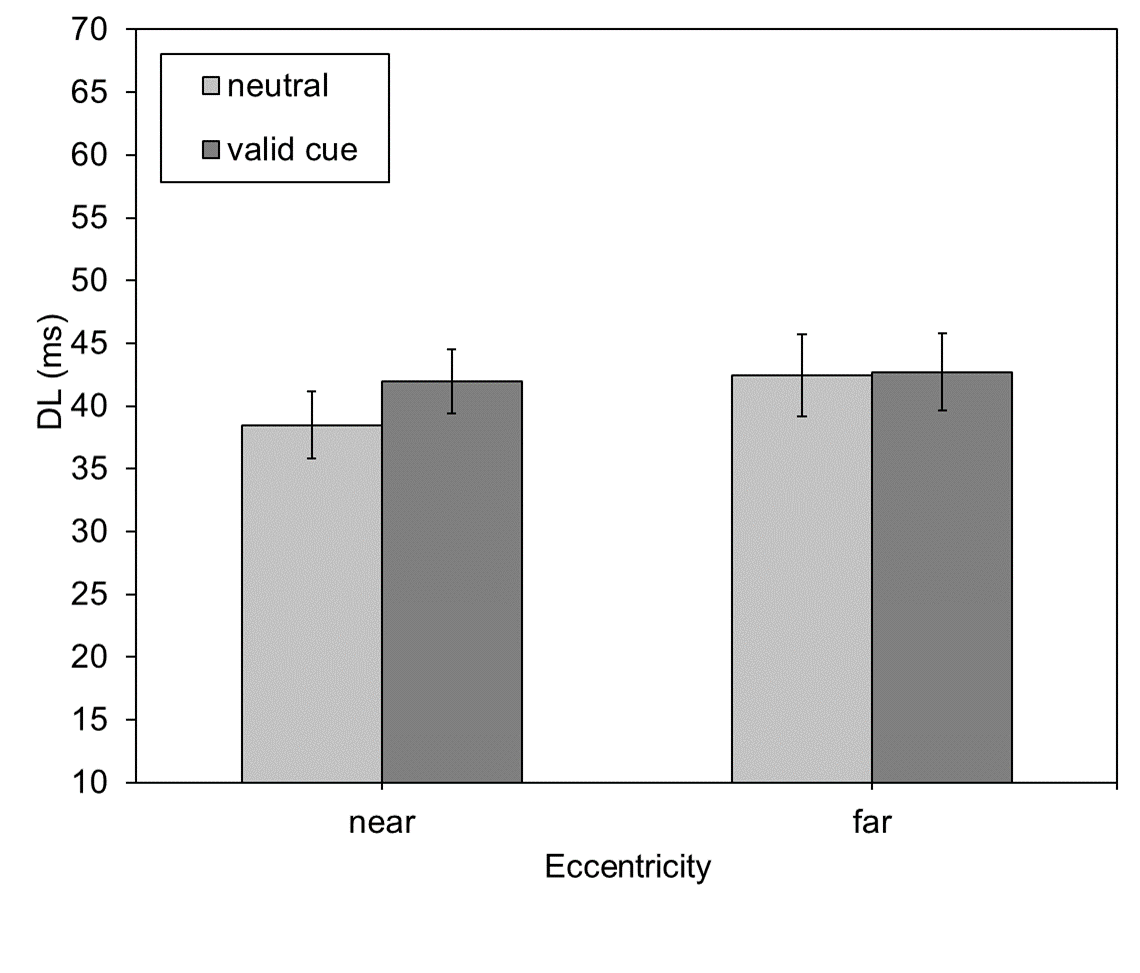
*

*Figure S1.* Mean DL values in milliseconds as a function of the eccentricity of the comparison stimulus (near, far) and the two cueing conditions (valid vs. neutral cue) in Experiment 1. Error bars represent the standard error (SE). *Note:* * *p* < .05, ** *p* < .01, *** *p* < .001.

## Analysis of the difference limen in Experiment 2

This appendix contains the analysis of the difference limen (DL) for *n* = 28 participants in Experiment 2. A 2 × 2 repeated measures ANOVA with cue (neutral, valid) and eccentricity (3°, 9°) as repeated measures factors was conducted on DL values using JASP 0.16.4.0 (JASP Team, 2023).

Mean DL values are depicted in Figure S2. For the neutral cue condition, mean DL values are *M_neutral/3°_* = 29.144 ms (*SE_neutral/3°_* = 2.031 ms) in 3° of eccentricity and *M_neutral/9°_* = 33.034 ms (*SE_neutral/9°_* = 2.274 ms) in 9° of eccentricity. For validly cued stimuli, mean DL values in 3° of eccentricity are *M_valid/3°_* = 29.121 ms (*SE_valid/3°_* = 2.146 ms) and *M_valid/9°_* =39.330 ms (*SE_valid/9°_* = 4.274 ms) for 9° of eccentricity. A repeated measures ANOVA revealed a significant main effect of eccentricity *F*(1,27) = 9.056, *p* = .006, $\eta_{p}^{2}$ = .251, yet no significant main effect of cue *F*(1,27) = 2.52, *p* = .124, $\eta_{p}^{2}$ = .085 and no significant interaction *F*(1,27) = 3.036, *p* = .093, $\eta_{p}^{2}$ = .101.

*
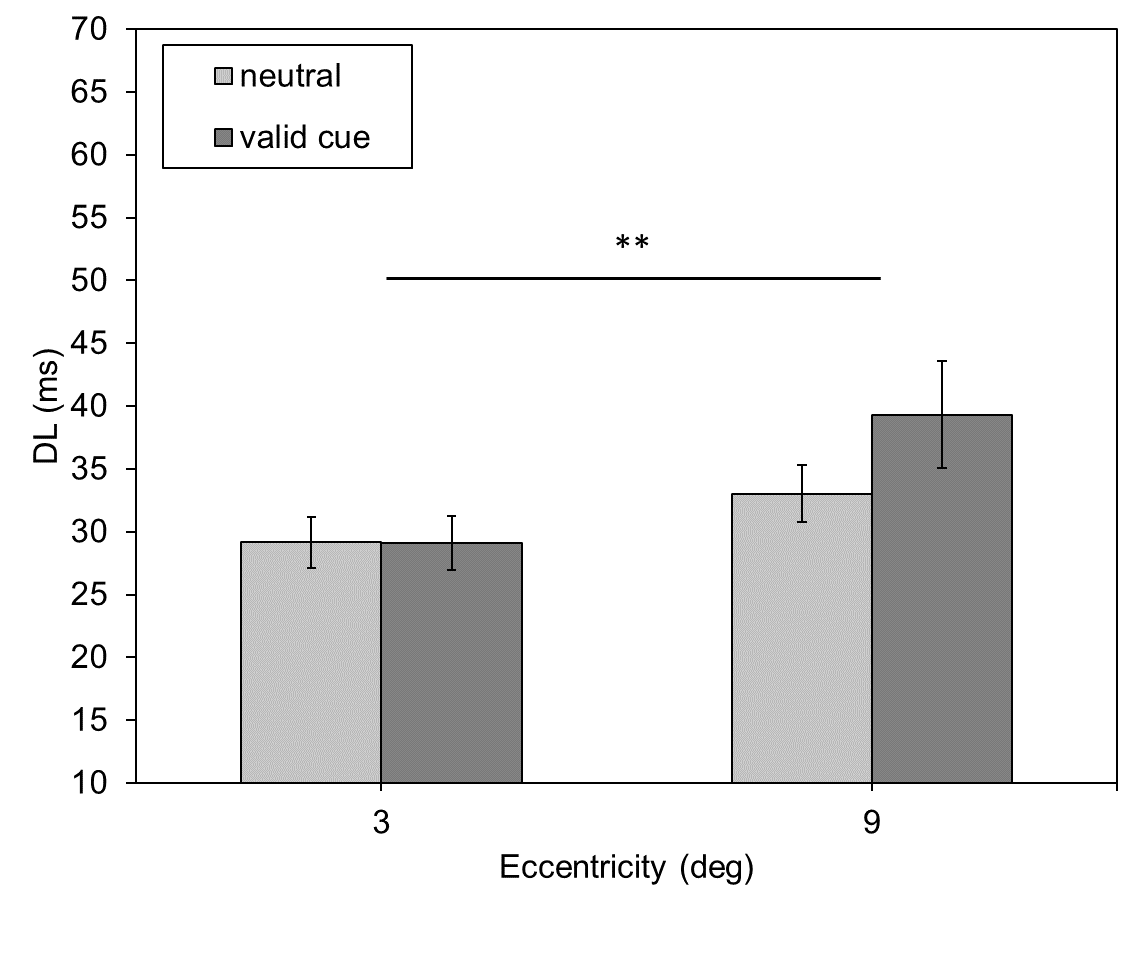
*

*Figure S2.* Mean DL values in milliseconds as a function of the eccentricity of the comparison stimulus (3°, 9°) and the two cueing conditions (valid vs. neutral cue) in Experiment 2. Error bars represent the standard error (SE). *Note:* * *p* < .05, ** *p* < .01, *** *p* < .001.
